# Protection of Retina by αB Crystallin in Sodium Iodate Induced Retinal Degeneration

**DOI:** 10.1371/journal.pone.0098275

**Published:** 2014-05-29

**Authors:** Peng Zhou, Ram Kannan, Christine Spee, Parameswaran G. Sreekumar, Guorui Dou, David R. Hinton

**Affiliations:** 1 Doheny Eye Institute, Los Angeles, California, United States of America; 2 Departments of Pathology, Keck School of Medicine of the University of Southern California, Los Angeles, California, United States of America; 3 Ophthalmology, Keck School of Medicine of the University of Southern California, Los Angeles, California, United States of America; Georgia Regents University, United States of America

## Abstract

Age-related macular degeneration (AMD) is a leading cause of blindness in the developed world. The retinal pigment epithelium (RPE) is a critical site of pathology in AMD and αB crystallin expression is increased in RPE and associated drusen in AMD. The purpose of this study was to investigate the role of αB crystallin in sodium iodate (NaIO_3_)-induced retinal degeneration, a model of AMD in which the primary site of pathology is the RPE. Dose dependent effects of intravenous NaIO_3_ (20-70 mg/kg) on development of retinal degeneration (fundus photography) and RPE and retinal neuronal loss (histology) were determined in wild type and αB crystallin knockout mice. Absence of αB crystallin augmented retinal degeneration in low dose (20 mg/kg) NaIO_3_-treated mice and increased retinal cell apoptosis which was mainly localized to the RPE layer. Generation of reactive oxygen species (ROS) was observed with NaIO_3_ in mouse and human RPE which increased further after αB crystallin knockout or siRNA knockdown, respectively. NaIO_3_ upregulated AKT phosphorylation and peroxisome proliferator–activator receptor–γ (PPAR_γ_) which was suppressed after αB crystallin siRNA knockdown. Further, PPAR_γ_ ligand inhibited NaIO_3_-induced ROS generation. Our data suggest that αB crystallin plays a critical role in protection of NaIO_3_-induced oxidative stress and retinal degeneration in part through upregulation of AKT phosphorylation and PPAR_γ_ expression.

## Introduction

Age-related macular degeneration (AMD) is characterized by progressive degeneration of the macular region of the retina resulting in loss of central vision. AMD is the leading cause of irreversible blindness in the developed world [1]. Clinically, AMD manifests in two forms; a non-exudative dry form and an exudative, neovascular wet form [1], [2]. Geographic atrophy (GA) is an advanced form of dry AMD with extensive atrophy and loss of the retinal pigment epithelium (RPE) and overlying photoreceptors and is responsible for 10–20% of cases of legal blindness from AMD [3], [4]. At present, there is no available effective treatment for GA.

A number of murine models have been generated that simulate features of dry AMD including RPE degeneration, lipofuscin accumulation, subretinal deposits, and loss of photoreceptors [5]–[12]. Our laboratory recently showed that bone morphogenetic protein-4 (BMP-4) is highly expressed in dry AMD and mediates oxidative stress-induced senescence in RPE in in vitro dry AMD thus serving as a molecular switch between atrophic and neovascular AMD [13], [14]. Localized RPE debridement or genetic ablation of RPE can lead to a profound reduction in RPE cells and consequent loss of photoreceptors [15]. Retinotoxicity can also be induced by endogenous and exogenous agents in laboratory animals. Mice receiving polyinosine-polycytidylic acid (Poly I: C) had morphological changes similar to that of humans with dry ARMD exhibiting soft and/or hard Drusen, GA [16]. Recently, the conditional ablation of the microRNA processing enzyme DICER1 was shown to induce RPE degeneration in mice [17]. Genetic or pharmacological inhibition of inflammasome components (NLRP3, MYD88) was reported to prevent RPE degeneration induced by DICER1 loss or AluRNA exposure [18]. While most of the animal models for GA mentioned above are long-term involving prolonged treatment regimens, the NaIO_3_-induced retinal degeneration model has proven to be a convenient and widely used model, because it is rapid, reproducible and has a primary site of pathology in the RPE [6], [19]–[22]. Thus, in the present study, we have utilized the NaIO_3_ model in 129S6/SvEvTac mice to study mechanisms of retinal degeneration.

Crystallins are members of the small heat shock protein (sHSP) family, and αB crystallin has been found to have high chaperone efficiency, and bind misfolded proteins with high affinity and stoichiometry [23]. An increased expression of αB crystallin was found in RPE and associated drusen in dry AMD [24], [25]. Both αA and αB crystallin are expressed in the mouse retina [26]–[28]. Mice lacking αB crystallin have provided considerable insights into the functional roles of this protein [29]. Our laboratory has shown that RPE cells from mice lacking αB crystallin are more susceptible to oxidative and endoplasmic reticulum stress as compared to wild type RPE [28], [30], [31]. Further we found that RPE cells overexpressing αB crystallin showed resistance to apoptosis, suggesting that αB crystallin may prevent stress-induced cell death [32]. Recently, evidence for the secretion of αB crystallin by RPE exosomes and protection of neighboring photoreceptors and RPE by exogenous αB crystallin was presented by our laboratory [33] suggesting that αB crystallin has significant potential in retinal therapy.

This study was undertaken to investigate the role of αB crystallin in a model of NaIO_3_ induced retinal degeneration in 129S6/SvEvTac mice. Further, using cultured mouse and human RPE cells, we also investigated the mechanism of regulation of cell death from NaIO_3_-induced oxidative stress by αB crystallin. Our major finding is that absence of αB crystallin in αB crystallin knockout mice causes more severe degeneration of the retina in NaIO_3_-treated mice as compared to wild type mice treated with NaIO_3_. Further, our studies also suggest that αB crystallin plays a critical role in protection of NaIO_3_-induced oxidative stress and retinal degeneration in part through upregulation of AKT phosphorylation and PPAR_γ_ expression.

## Results

### Selection of optimal *in vivo* dose and duration of NaIO_3_ treatment

**Figure 1:**
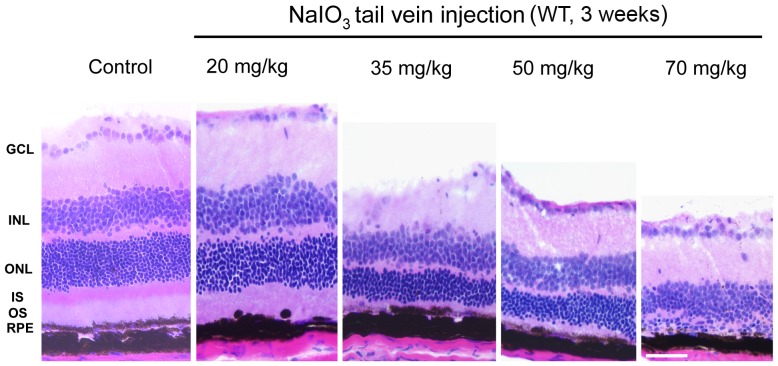
H & E staining of retina showing dose dependent effect of NaIO_3_ in 129S6/SvEvTac wild type (WT) mice. The intravenous NaIO_3_ doses used were 20, 35, 50 and 70 mg/kg body weight. Mice were euthanized after 3 weeks of treatment, eyes were enucleated, frozen sections of retinal were obtained, and H & E staining was performed. Most mice treated with 20 mg/kg showed an intact layer of contiguous RPE although occasional mice showed focal RPE degeneration and loss at the end of 3 weeks (as shown in this figure). The higher doses showed moderate and severe damage to the RPE cell layer, and outer nuclear layer. GCL: ganglion cell layer; ONL: outer nuclear layer; INL: inner nuclear layer; IS: inner segment; OS: outer segment. Bar equals 75 µm.

Preliminary experiments were performed to select an optimal dosage of NaIO_3_ that was used in all subsequent *in vivo* experiments in mice. We tested the effect of a single intravenous injection of 20, 35, 50 and 70 mg/kg NaIO_3_ for 1, 2 and 3 weeks on retinal morphology. The histologic data from varying doses of NaIO_3_ treatment are presented in Figure 1. NaIO_3_-induced retinal degeneration increased with the dose. The extent of degeneration was absent to mild with 20 mg/kg, moderate with 35 and 50 mg/kg and was severe with 70 mg/kg dose 3 week after NaIO_3_ treatment (Figure 1). We chose the 20 mg/kg NaIO_3_ dose that produced no more than mild degeneration in all our subsequent experiments to enable studying the exacerbating effects of αB crystallin knockout on retinal damage (see below). While high doses of NaIO_3_ resulted in retinal degeneration as early as 1 week post-injection, the low dose (20 mg/kg) NaIO_3_ showed damage localized to the RPE at the 3 week time-point (Fig S1).

### Fundus photography shows accelerated NaIO_3_-induced retinal degeneration in αB crystallin knockout mice

**Figure 2:**
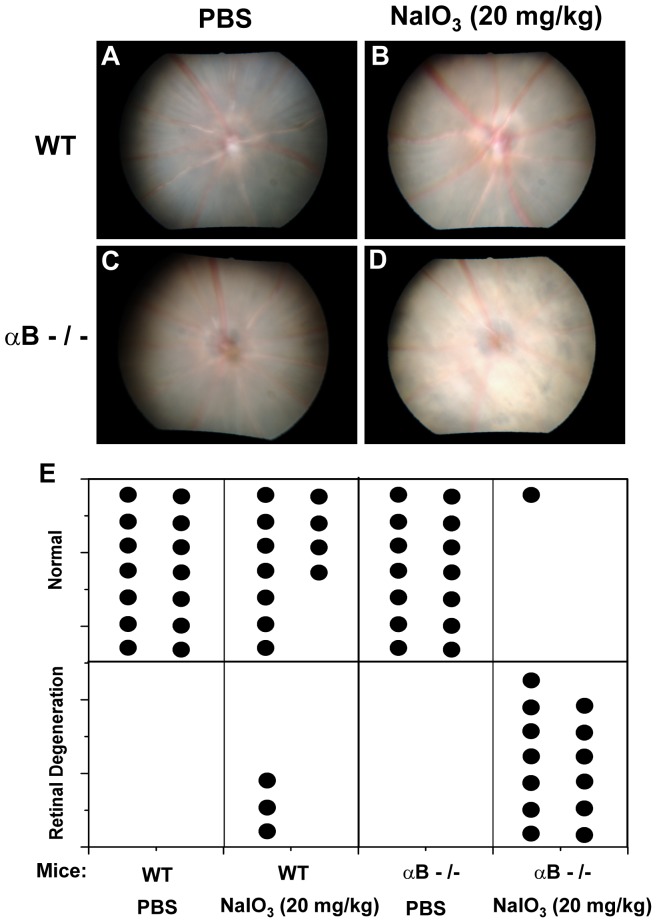
Fundus photograph of control and NaIO_3_-treated αB crystallin knockout mice (αB-/-). Three weeks after tail vein injection of 20/kg NaIO_3_, fundus photograph was taken in PBS-treated wild type (WT) (A), NaIO_3_-treated WT (B), PBS-treated αB-/- (C), and NaIO_3_-treated αB-/- (D) mice. Arrows indicate sites of retinal degeneration. Thirteen NaIO_3_-treated αB-/- mice showed patchy retinal degeneration three weeks after NaIO_3_ injection (E). Only three out of fourteen eyes of NaIO_3_-treated WT mice showed retinal degeneration (E). A statistically significant difference was found between NaIO_3_-treated αB-/- and NaIO_3_-treated WT mice (P<0.001).

To determine the extent of NaIO_3_-induced retinal degeneration, we compared the fundus photographs of mice from PBS-treated WT, NaIO_3_-treated WT, PBS-treated αB crystallin knockout, and NaIO_3_-treated αB crystallin knockout groups at the end of 3 weeks. The dose of NaIO_3_ in these studies was 20 mg/kg. The retinal degeneration induced by NaIO_3_ in mice appeared as patchy white retinal lesions when observed by fundus photography (Fig. 2).

The fundus photographs of thirteen out of fourteen NaIO_3_-treated αB crystallin knockout mice showed patchy retinal degeneration three weeks after injection (Fig.2D, E). Only three out of fourteen eyes of NaIO_3_-treated wild type mice showed retinal degeneration (Fig 2B, E). Thus, the difference in the number of mice with retinal degeneration between NaIO_3_-treated αB crystallin knockout mice and NaIO_3_-treated wild type mice was highly significant (P<0.001). No apparent degeneration could be seen in control, untreated wild type or αB crystallin knockout retina.

### Histopathology shows accelerated NaIO_3_-induced degeneration in αB crystallin knockout mice

**Figure 3:**
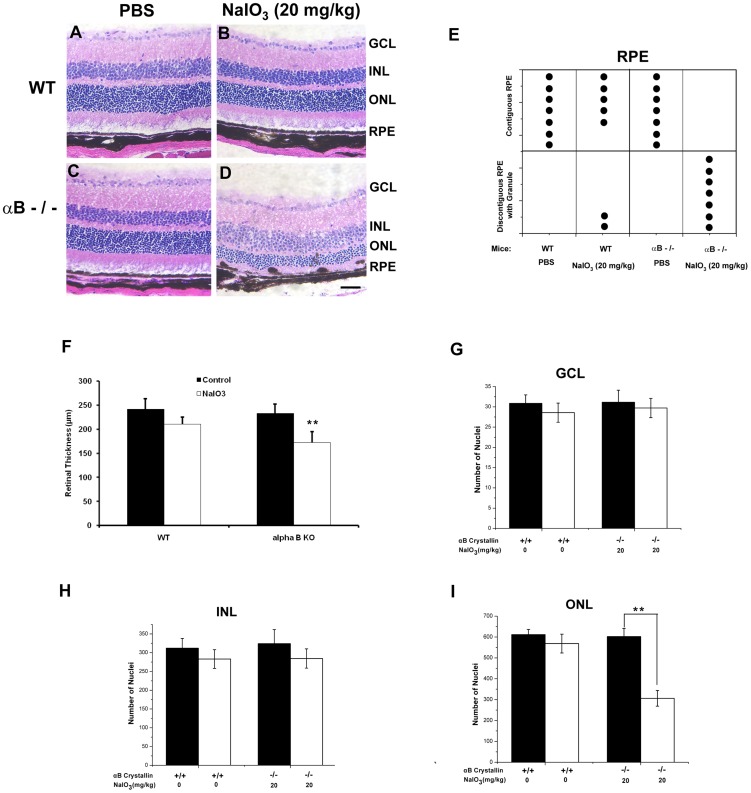
Histopathology of retina from control and NaIO_3_-treated αB crystallin knockout (αB-/-) mice. Three weeks after tail vein injection of 20/kg NaIO_3_, eyes were enucleated and frozen sections were stained with H&E. The four experimental groups of mice were PBS-treated WT (A), NaIO_3_-treated WT (B), PBS-treated αB-/- (C), and NaIO_3_-treated αB-/- (D). The RPE layer in αB-/- mice with NaIO_3_ injection were discontiguous and damaged. Only two out of seven WT mice showed discontiguous and damaged RPE (E). Bar graph showing retinal thickness (µm) with and without NaIO_3_ in WT and αB-/- mice. (F). Retinal thickness was significantly lower (P<0.01) in NaIO_3_ treated αB-/- mice when compared to the corresponding WT group. No significant differences in the number of nuclei in the ganglion cell layer, outer nuclear layer or inner nuclear layer were found between the NaIO_3_-injected and PBS-injected WT mice (G–H). The number of nuclei per unit area in the outer nuclear layer of NaIO_3_ injected αB-/- mice showed a significant decrease (P<0.01) as compared to control without NaIO_3_ injection (I). RPE: RPE cell layer; ONL: outer nuclear layer; INL: inner nuclear layer; GCL: ganglion cell layer. Data are mean ± SEM, n = 7/group, **P<0.01. Bar equals 75 µm.

The primary site of pathology after NaIO_3_ injection (20 mg/kg) was the RPE layer; we observed that the RPE layer was discontiguous and damaged in all αB crystallin knockout mice, while only two out of seven wild type mice showed these changes in the RPE (Fig. 3). Using TUNEL staining we confirmed that with NaIO_3_ (20 mg/kg; 3 week time point) cell death was localized to the RPE layer in the αB crystallin knockout mice (Figure S2). Significant differences were found between NaIO_3_-treated wild type mice and NaIO_3_-treated αB crystallin knockout mice in the extent of RPE degeneration (P<0.01). Retinas from αB crystallin knockout mice (Fig. 3D) revealed more severe degeneration from NaIO_3_ injection as compared to wild-type retinas (Fig. 3B). Total retinal thickness was significantly decreased (P<0.01) in αB crystallin knockout mice with NaIO_3_ treatment as compared to untreated αB crystallin knockout group (P<0.01). In contrast, in wild type mice, no significant difference in retinal thickness after treatment with NaIO_3_ was found vs. untreated controls. (Fig. 3F). An assessment of the localization of retinal damage by NaIO_3_ was made by counting the number of nuclei in the inner nuclear layer (INL), outer nuclear layer (ONL) and ganglion cell layer (GCL) of wild type and αB crystallin knockout retina (Fig. 3G–I). This analysis revealed that the loss of nuclei was more prominent at 3 weeks post-NaIO_3_ injection in αB crystallin knockout retina vs. that of wild type. The number of nuclei per unit area showed a significant decrease with NaIO_3_ injection in the ONL of αB crystallin knockout mice which was statistically significant (P<0.01; Fig. 3I). No significant differences in the number of nuclei in any of the other nuclear layers (GCL, INL) were found between the NaIO_3_-injected and PBS-injected groups of wild type mice (Fig. 3-G,H).

### Reduced ERG amplitudes in NaIO_3_-treated αB crystallin knockout mice

**Figure 4:**
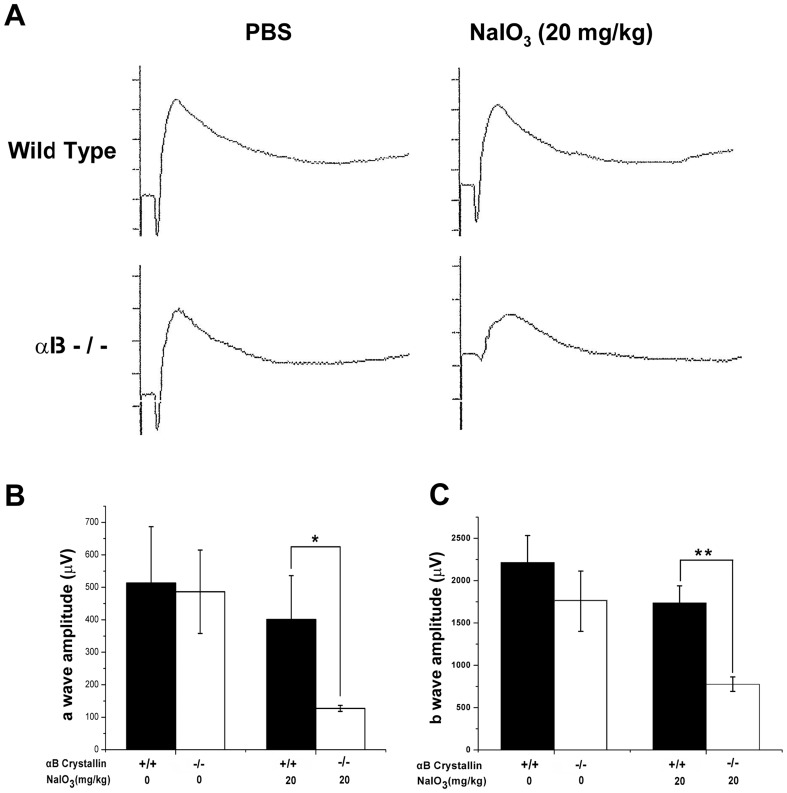
Reduced ERG amplitudes in NaIO_3_-treated αB crystallin knockout (αB-/-) mice. Three weeks after tail vein injection of 20/kg body weight NaIO_3_, mesopic ERG responses were recorded in PBS-treated WT, NaIO_3_-treated WT, PBS-treated αB-/-, and NaIO_3_-treated αB-/- mice (representative ERGs shown in A). The amplitudes of *a* wave of the ERG of NaIO_3_-treated αB-/- mice decreased by 68.3% compared with that of PBS-treated αB-/- mice (B). The amplitudes of *b* wave of the ERG of NaIO_3_-treated αB-/- mice decreased by 55.3%, compared with that of PBS-treated αB-/- mice (C). Data are mean ± SEM, n = 5/group, *P<0.05, **P<0.01.

To determine whether the absence of αB crystallin had an effect on the retinal function of NaIO_3_-treated mice, we compared mesopic (mixed rod and cone) ERG responses. These studies to assess the functional response of neural retina were performed in four groups of mice (PBS-treated WT, NaIO_3_-treated WT, PBS-treated αB crystallin knockout, and NaIO_3_-treated αB crystallin knockout) that received a dose of 20 mg/kg NaIO_3_ at the end of 3 weeks. Significant differences were observed in the ERGs of NaIO_3_-treated αB crystallin knockout mice compared with the PBS-treated αB crystallin knockout mice (Fig 4A). The amplitude of the *a* wave of the ERG, that originates from the photoreceptors, of NaIO_3_-treated αB crystallin knockout mice decreased by 68.3% compared with that of PBS-treated αB crystallin knockout mice (Fig 4B). The amplitude of the *b* wave of the ERG, (that originates from the bipolar cells), of NaIO_3_-treated αB crystallin knockout mice decreased by 55.3% compared with that of PBS-treated αB crystallin knockout mice (Fig 4C). No significant differences were found between the ERGs of the NaIO_3_-treated and control wild type mice at this low dose (Fig 4B,C).

### Increased production of reactive oxygen species (ROS) in αB crystallin knockout RPE and cultured human RPE cells transfected with αB crystallin siRNA after NaIO_3_ treatment

**Figure 5:**
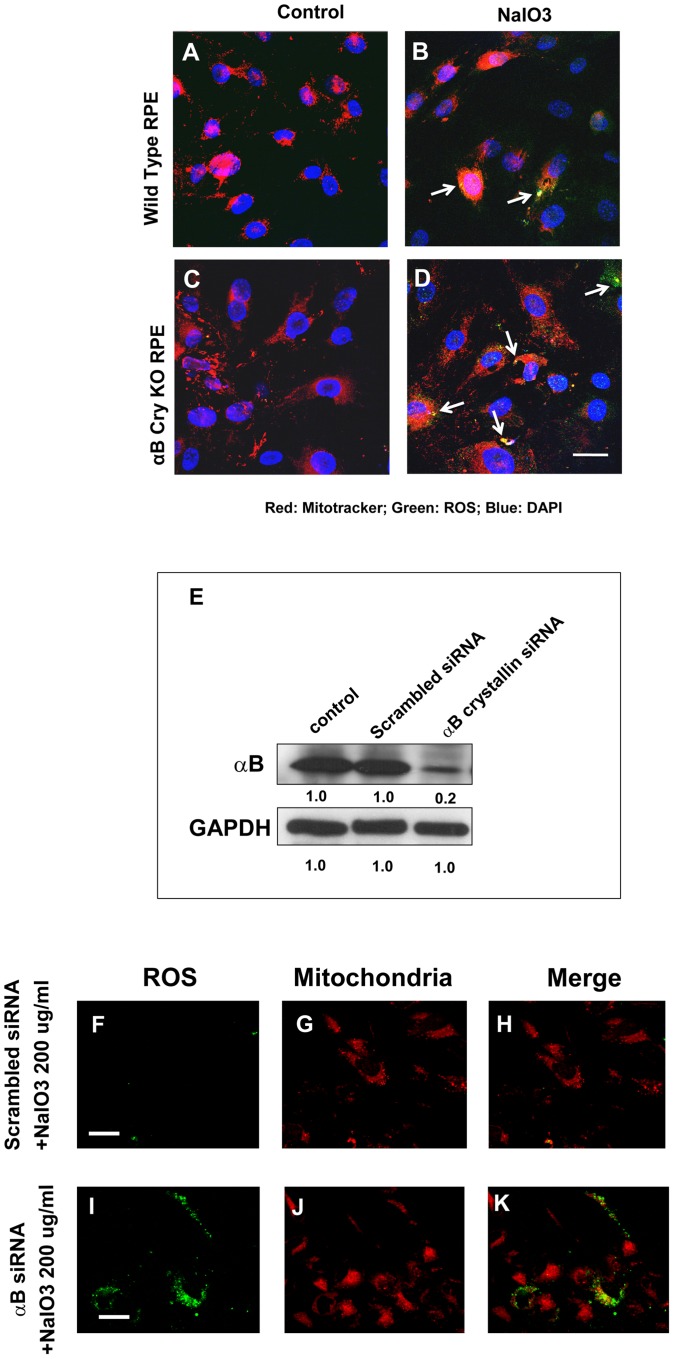
Increased production of reactive oxygen species (ROS) in mouse primary RPE cells and human RPE cells transfected with αB crystallin (αB-/-) siRNA after NaIO_3_ treatment. Confluent mouse RPE cells from αB-/- and WT mice were treated with 200 µg/ml NaIO_3_ for 24 h. In panels A–D, DAPI is shown in blue, ROS staining in green and mitotracker in red. ROS staining was observed in WT RPE cells treated with NaIO_3_ (A–B). RPE cells from αB-/- mice (C–D, see white arrows) showed increased accumulation of ROS that partially colocalized with mitochondria. For studies with human RPE cells, αB crystallin was knocked down (∼80%) by siRNA transfection (Fig. 5E). Increased ROS production was observed in αB crystallin si-RNA transfected human RPE cells with NaIO_3_ as compared to scrambled siRNA transfected cells which showed negligible ROS staining (I–K). (F–H). Scale bars represent 20 µm for A–D and 10 µm for F–K, respectively.

These experiments were performed in both mouse and human RPE cultured in 0.5% FBS-containing DMEM; cells were treated with 200 µg/ml NaIO_3_ for 24 h. Treatment with NaIO_3_ induced ROS production in RPE from WT mice which was not found in untreated controls (Fig. 5A–B). ROS partially co-localized with mitochondria. The ROS production was even higher in αB crystallin knockout RPE after NaIO_3_ treatment (arrows, Fig. 5D). Negligible ROS was produced in αB crystallin knockout RPE without NaIO_3_ (Fig.5C). To further evaluate the effect of NaIO_3_ on ROS production, we studied primary human RPE cells after αB crystallin knockdown. The percentage of knockdown of αB crystallin in human RPE by siRNA transfection was about 80% as determined by Western blot analysis (Fig 5E). Treatment with NaIO_3_ resulted in a pronounced intracellular generation of ROS that was predominantly localized to the mitochondria in αB crystallin siRNA-transfected RPE cells (Fig. 5I–K). However, the staining for ROS was much less prominent in NaIO_3_-treated RPE cells with scrambled siRNA (Fig. 5F–H). Thus, these results show that knockout or siRNA knockdown of αB crystallin results in increased generation of ROS in RPE cells treated with NaIO_3_.

### Mode of cell death in RPE exposed to low dose of NaIO_3_ is not by necrosis

**Figure 6:**
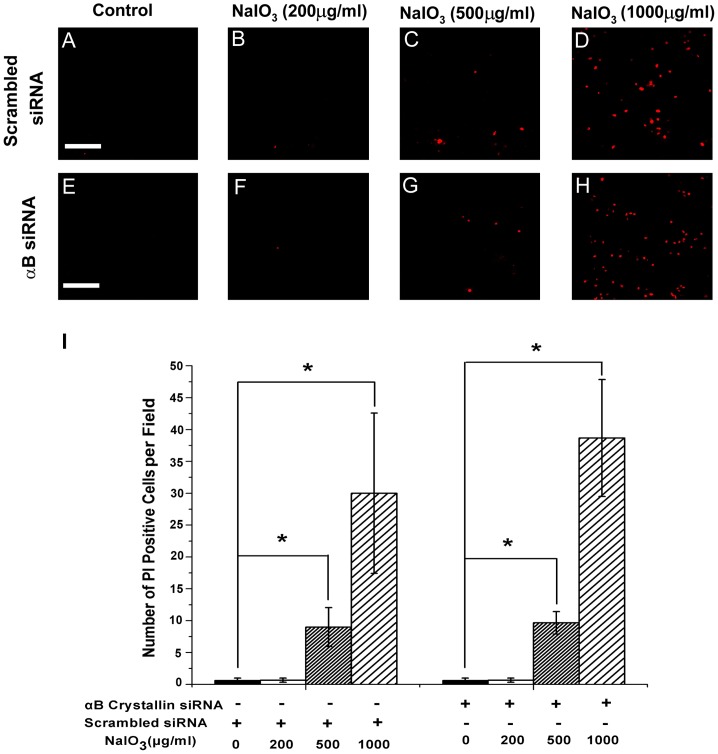
Occurrence of necrosis with high doses of NaIO_3_ in human RPE cells. Forty-eight hours after scrambled siRNA or αB crystallin siRNA transfection, RPE cells were treated with 200, 500, 1000 µg/ml NaIO_3_ for 24hours. Propidium iodide (PI) staining positive cells were determined in scrambled siRNA-transfected RPE cells (A–D) and αB crystallin siRNA-transfected human RPE cells (E–H) treated with NaIO_3_. Treatment with 500 and 1000 µg/ml of NaIO_3_ resulted in increased PI positive cells both in control RPE cells and αB crystallin siRNA pretreated human RPE cells (I). However, no significant differences were found between the number of PI positive cells in control RPE groups and αB crystallin siRNA-transfected group (I). Data are mean ± SEM from three individual experiments, αB siRNA refers to αB crystallin siRNA. *P<0.05. Scale bar = 40 µm.

Propidium iodide (PI) staining was performed to assess necrotic features in RPE cells incubated with different doses of NaIO_3_. The NaIO_3_ treatment of RPE was performed for 24 h in 0.5% FBS-containing DMEM at doses of 200, 500, or 1000 µg/ml, respectively. Confocal microscopy images are presented in Fig. 6 (A–H) that show PI staining in control (scrambled siRNA) RPE nuclei (Fig. 6A–D) and αB crystallin siRNA-transfected RPE nuclei (Fig. 6E–H) with or without NaIO_3_ treatment. No significant differences were found in the number of PI positive cells between control group and the group treated with 200 µg/ml of NaIO_3_ in both scrambled siRNA and αB crystallin siRNA-transfected human RPE cells. Treatment with 500 and 1000 µg/ml of NaIO_3_ resulted in increased PI positive cells both in scrambled siRNA RPE and αB crystallin siRNA pretreated RPE (P<0.01). However, no significant differences were found between the number of PI positive cells in control RPE and αB crystallin siRNA-transfected groups (Fig. 6I). Therefore, it can be concluded that high dose NaIO_3_ (500 µg/ml and 1000 µg/ml) induced predominantly RPE cell necrosis, while induction of necrosis was negligible or insignificant with low dose NaIO_3_ (200 µg/ml).

### Increased apoptosis in αB crystallin siRNA-transfected RPE with low dose NaIO_3_

**Figure 7:**
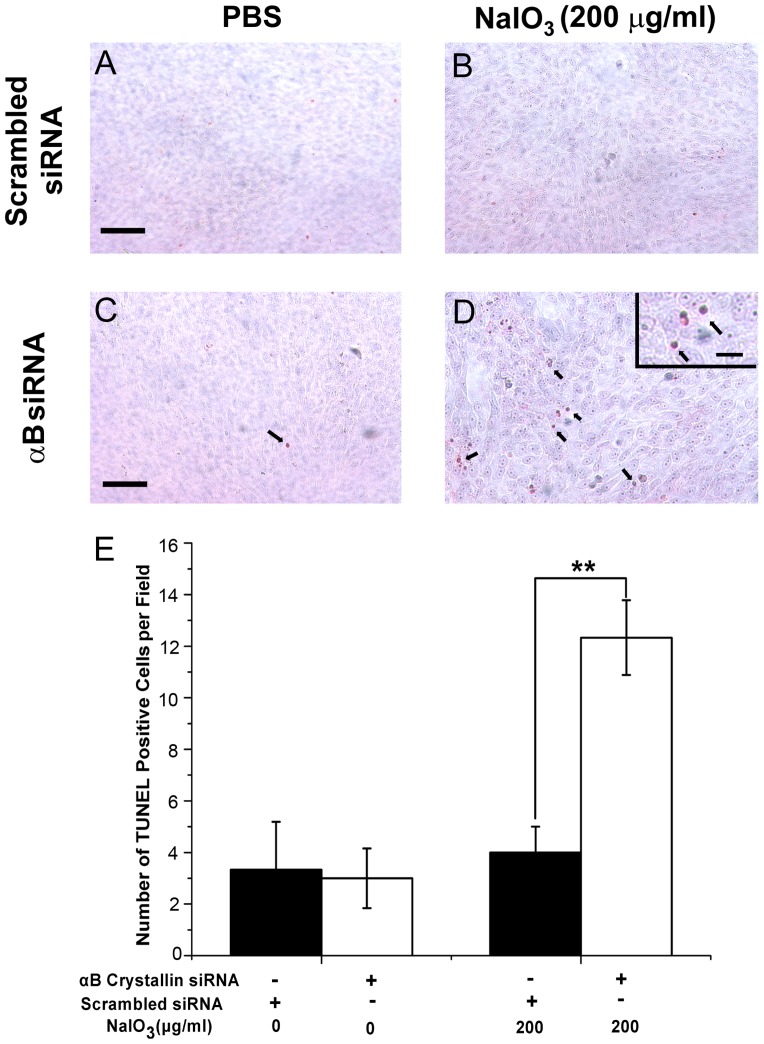
Induction of apoptosis in RPE cells with low dose of NaIO_3_. Forty-eight hours after scrambled siRNA or αB crystallin siRNA transfection, human RPE cells were treated with 200 µg/ml NaIO_3_ for 24 hours. TUNEL staining was performed in scrambled siRNA-transfected human RPE cells (A, B) and αB crystallin siRNA-transfected human RPE cells (C, D). Treatment with NaIO_3_ resulted in increased TUNEL-positive cells with αB crystallin siRNA-transfection as compared with scrambled siRNA controls. (arrows in inset in D and E). Data are mean ± SEM from three individual experiments, αB siRNA refers to αB crystallin siRNA. **P<0.01. Scale = 40 µm for A–D and 10 µm for inset in D.

TUNEL staining was performed to assess the extent of apoptosis with NaIO_3_ in RPE cells. Fig. 7 shows TUNEL staining in control RPE cells (Fig. 7A–B) and αB crystallin siRNA-transfected RPE cells (Fig. 7C–D) treated with a low dose (200 µg/ml) of NaIO_3_. The duration of NaIO_3_ exposure of RPE in 0.5% FBS-containing DMEM was 24 h. Treatment with 200 µg/ml NaIO_3_ resulted in increased TUNEL-positive cells with αB crystallin siRNA-transfection as compared with scrambled siRNA controls (Fig. 7E) (P<0.01). However, no significant difference was found between control and NaIO_3_-treated RPE cells without αB crystallin siRNA-transfection. Thus NaIO_3_ induces apoptosis in αB crystallin siRNA-transfected RPE cells that were exposed to low doses.

### Increased caspase 3 activation with NaIO_3_ and αB crystallin siRNA

**Figure 8:**
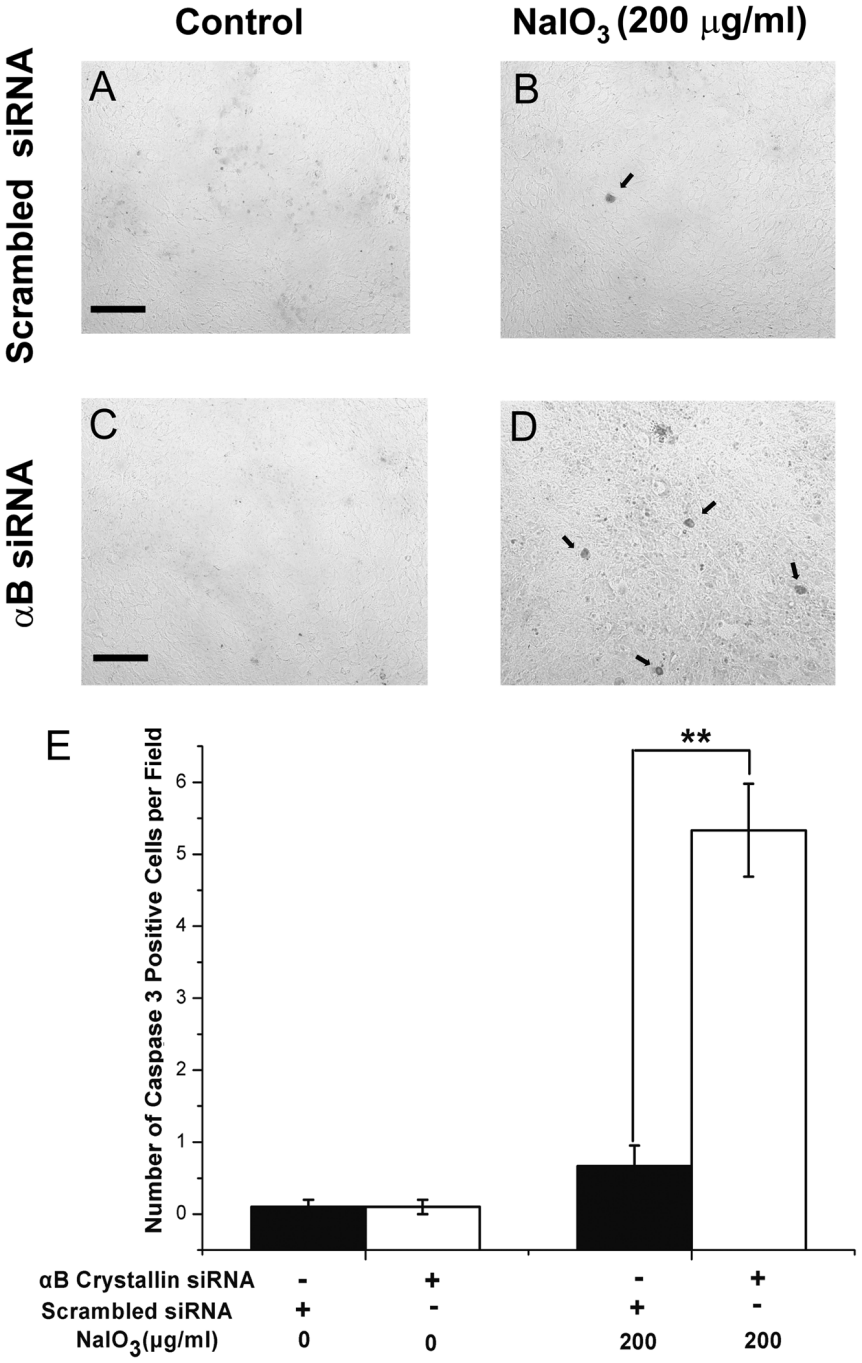
Caspase 3 activation with NaIO_3_ treatment and increased activation with knockdown of αB crystallin siRNA in human RPE cells. Forty-eight hours after scrambled siRNA or αB crystallin siRNA transfection, human RPE cells were treated with 200 µg/ml NaIO_3_ for 24 hours. Cleaved caspase 3 staining was performed in scrambled siRNA-transfected human RPE cells (A, B) and αB crystallin siRNA-transfected human RPE cells (C, D). Treatment with 200 µg/ml of NaIO_3_ resulted in increased amount of cleaved caspase 3-positive cells with αB crystallin siRNA-transfection vs. scrambled siRNA controls (E). Data are mean ± SEM from three individual experiments, αB siRNA refers to αB crystallin siRNA in panels C and D. **P<0.01. Scale bar = 40 µm.

Cleaved caspase 3 staining was performed to confirm that the mechanism of cell death was by apoptosis. Fig. 8 shows immunostaining of cleaved caspase 3 in control human RPE cells (Fig. 8A–B) and αB crystallin siRNA-transfected RPE cells (Fig. 8C–D) treated with low dose NaIO_3_. The duration and dose of NaIO_3_ exposure of RPE in 0.5% FBS-containing DMEM was 24 h and 200 µg/ml. Treatment of RPE cells with 200 µg/ml NaIO_3_ resulted in an increase in the number of cleaved caspase 3-positive cells with αB crystallin siRNA-transfection as compared to scrambled siRNA controls (Fig. 8E) (P<0.01). However, no significant difference was found between control and NaIO_3_ treated RPE cells without αB crystallin siRNA-transfection. This indicates that apoptosis with low dose NaIO_3_ (200 µg/ml) in αB crystallin siRNA-transfected RPE cells occurs via caspase 3 activation.

### Signaling molecules associated with NaIO_3_-induced RPE cell death

**Figure 9:**
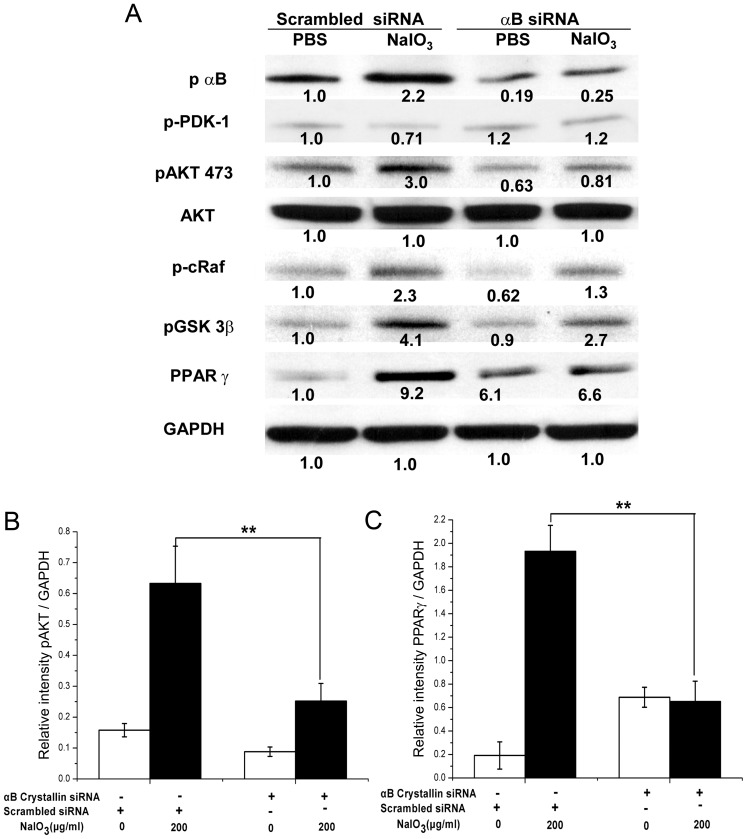
Western blot analysis showing changes in expression of signaling molecules with NaIO_3_. Forty-eight hours after scrambled siRNA or αB crystallin siRNA transfection, human RPE cells were treated with 200 µg/ml NaIO_3_ for 24 hours. Cells were lysed and western blot was performed. (A) Phosphorylated level of AKT at serine 473 increased after the treatment of low dose (200 µg/ml) NaIO_3_ (B). The increase of phospho-AKT was lower in αB crystallin siRNA-transfected RPE cells treated with NaIO_3_. Similar trends were found for phospho-GSK 3β and phospho-c-Raf, and PPAR_γ_ and quantification is shown for PPAR_γ_ in (C). However, no significant changes were found in phospho-PDK1, a kinase upstream of AKT. All experiments were performed three times and representative gels are presented. Protein expression levels were measured by Bandscan software, normalized to GAPDH, and labeled under each corresponding band. The bars for group B and C for pAKT and PPAR_γ_ are derived from 3 individual experiments. αB siRNA refers to αB crystallin siRNA. pαB in panel A refers to phosphorylated αB crystallin. **P<0.01.

We investigated the mechanism of the exacerbation of NaIO_3_-induced RPE cell apoptosis in the absence of αB crystallin by analyzing several apoptotic signaling proteins (Fig. 9). Expression of phosphorylated AKT at serine 473 increased after treatment with low dose (200 µg/ml) NaIO_3_ (Fig. 9B). However, the increase of phospho-AKT was much lower in αB crystallin siRNA-transfected RPE cells treated with NaIO_3_ compared to controls. A similar trend was observed for phospho-GSK 3β and phosphorylated -c-Raf, signaling proteins downstream of AKT. Low dose NaIO_3_ markedly increased PPAR_γ_ expression in scrambled transfected RPE cells, while this increase could not be seen in αB crystallin siRNA-transfected RPE cells (Fig. 9C). However, no significant changes were evident with NaIO_3_ treatment in phospho-PDK1, a kinase upstream of AKT (Fig. 9A). This suggests that AKT may be the point of interaction with NaIO_3_ in the AKT signaling pathway (Fig. 9A).

**Figure 10:**
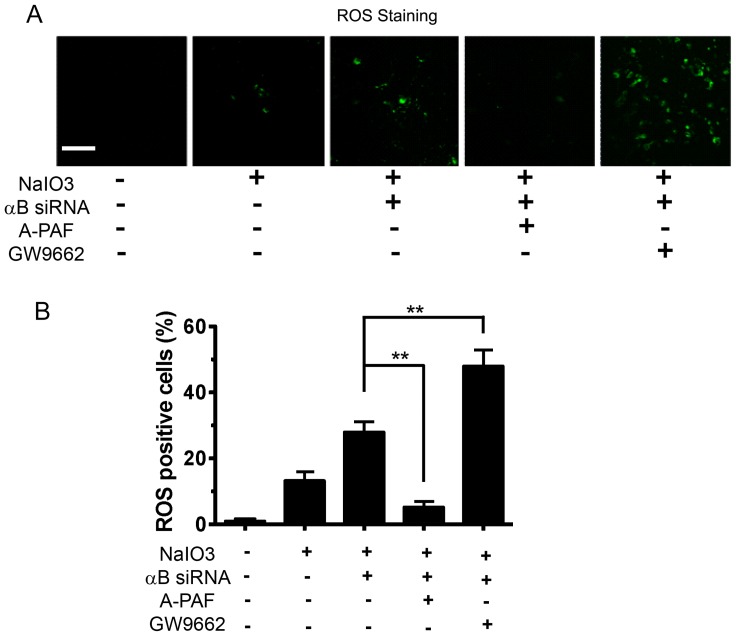
Detection of reactive oxygen species (ROS) in the presence and absence of PPAR_γ_ ligand/antagonist. Increased production of ROS was observed in RPE cells transfected with αB crystallin siRNA upon NaIO_3_ treatment as compared to control group. A-PAF, a PPAR_γ_ ligand, suppressed ROS production significantly (P<0.01) in αB crystallin siRNA transfected cells treated with NaIO_3_ (A). On the other hand, GW9662, a PPAR_γ_ antagonist significantly increased ROS positive cells in αB crystallin siRNA transfected cells treated with NaIO_3_. ROS positive cells were quantified from four random images derived from 4 individual experiments. αB siRNA refers to αB Crystallin siRNA. (B). Scale bar = 40 µm. **P<0.01.

The treatment with low dose (200 µg/ml) NaIO_3_ increased PPAR_γ_ expression in RPE. The increased expression of PPAR_γ_ possibly exerts a reactive protective role, as we observed A-PAF, a PPAR_γ_ ligand, significantly decreased the generation of ROS (Fig. 10A). On the other hand, GW9662, a PPAR_γ_ antagonist, caused a significant increase in NaIO_3_-induced production of ROS (P<0.01; Fig. 10B). The increase in PPAR_γ_ by NaIO_3_ was attenuated in NaIO_3_-treated αB crystallin siRNA-transfected RPE cells thereby compromising the protective defense by PPAR_γ_ under these conditions (Fig. 10A, B).

## Discussion

In an attempt to understand the protective role of αB crystallin in stress-induced RPE degeneration, we have investigated the effect of suppression of αB crystallin on apoptosis and have studied the signaling mechanisms associated with this phenomenon. For this purpose, we used a murine model of NaIO_3_-induced retinal degeneration *in vivo* and cell death in human RPE *in vitro*.

NaIO_3_ has been previously shown to induce selective degeneration of the RPE and consequent retinal degeneration [6], [19], [20]. A low dose of NaIO_3_ was used to induce retinal degeneration in this study. No significant retinal degeneration was found on fundus photography in wild type mice three weeks after tail vein injection of low dose NaIO_3_, while 93.7% (15/16) eyes of αB crystallin knockout mice exhibited retinal degeneration after the same treatment. A much higher dose and duration of treatment (100 mg/kg NaIO_3_; 6 weeks) was required in ICR strain of mice to induce changes in morphology in the retina [21] indicating that differences among mouse strains can also play a role [19]–[21]. Furthermore, previous ultrastructural and TUNEL labeling studies showed that RPE cell death induced by 100 mg/kg NaIO_3_ was from necrosis and that of the photoreceptors was from apoptosis [21]. In the present study, increased RPE apoptosis was found in low dose NaIO_3_-treated RPE cells from αB crystallin knockout mice and in human RPE after αB crystallin knockdown; however, necrosis was minimal. Necrotic cells increased in a dose dependent manner with high dose of NaIO_3_. Therefore, we may conclude that low dose of NaIO_3_ induces RPE cell apoptosis, while high dose of NaIO_3_ results in RPE cell necrosis.

The role of αB crystallin in cellular protection is becoming increasingly important because αB crystallin acts on a variety of cellular processes [23], [27]. Newer studies have taken advantage of αB crystallin's antiapoptotic and anti-inflammatory properties in devising therapy [34], [35]. For example, intravenous administration of αB crystallin in mice was found to reduce inflammation and thus play a protective role in experimental autoimmune demyelination [34], [36]. αB crystallin may play an important role in protection of retinal neurons from damage by metabolic and environmental stress as seen by evidence of elevated crystallin expression in light damaged photoreceptors and in models of retinal degeneration [37], [38]. αB crystallin could be important in the development of, or in response to, AMD since αB crystallin was found to be accumulated in RPE, drusen and Bruch membrane tissues from AMD patients [23], [24]. In a recent study, we found that αB crystallin is secreted via exosomes by RPE cells and presented evidence for its extracellular function in protecting neighboring RPE cells and photoreceptors from oxidative injury [33].

In our present studies, we found that lack of αB crystallin accelerated and augmented the retinal degeneration in NaIO_3_-treated mice *in vivo* and was associated with increased RPE cell apoptosis *in vitro*. In previous studies from our laboratories, we had reported that lack of αB crystallin renders RPE cells more susceptible to apoptosis from oxidative stress induced by H_2_O_2_ [28]. Similarly, when RPE cells were exposed to ER stress, apoptosis ensues and αB crystallin regulated ER-stress induced cell death [31]. Silencing of αB crystallin by siRNA knockdown exacerbated apoptosis while overexpression attenuated apoptotic cell death in RPE cells [30], [31]. Our present data show that induction of apoptosis by NaIO_3_ occurs through generation of ROS consistent with the known oxidative properties of iodate ions involving mitochondria [6], [19], [20].

In the αB crystallin knockout mouse, knockout of the αB crystallin gene also disrupted the closely related gene HSPB2 [29]. However, while HSPB2 is expressed in muscle tissues, our previous work established that HSPB2 is not expressed in normal or pathologic murine posterior eye cups, including the retina and RPE [29], [39]. Therefore, loss of HSPB2 has no effect on the evaluation of αB crystallin knockout in studies of retinal degeneration.

It was reported that there was no apparent phenotype in the retina of αB crystallin knockout mice [29]; however, our recent studies found that while the histology of the neural retina was unaffected, there was a mild decrease in retinal vessel density in the inner plexiform layer in αB crystallin knockout mice compared to wild type [39]. We found that absence of αB crystallin accelerated and augmented the degeneration of the retina in NaIO_3_ treated mice in this study. Further, apoptosis was exacerbated in RPE after αB crystallin siRNA knockdown. For example, we observed increased production of ROS in RPE cells from αB crystallin knockout mice and human RPE cells transfected with αB crystallin siRNA upon NaIO_3_ treatment. These results suggest that while an apparent retinal phenotype could not be found *in vivo* in normal conditions, suppression of αB crystallin does indeed cause injury and death at a cellular level in RPE after oxidative stress. Furthermore, the mode of cell death was via apoptosis and necrosis was not seen in RPE cells treated with low NaIO_3_ doses.

It is of interest that very recently it was shown that knockout of αA crystallin also exacerbates retinal degeneration in the NaIO_3_ model [40]. We have previously shown that αB crystallin is expressed at much higher levels in RPE than αA crystallin but that knockout of either αA or αB crystallin in RPE renders them more susceptible to oxidative stress [28]. Thus, it might be interesting to study the effects of double knockout of αA and αB crystallin on the extent of retinal degeneration in this model.

We used human RPE cultures *in vitro* to elucidate the mechanism of NaIO_3_ induced apoptosis under conditions of αB crystallin deficiency. PPAR-_γ_, a member of a nuclear receptor superfamily, plays a key role in numerous cellular functions and is a key regulator of mitochondrial biogenesis and of ROS metabolism [41]. We found that the expression of PPAR_γ_ protein in RPE cells increased after treatment with low dose NaIO_3_. The increase in PPAR_γ_ expression was significantly lower in αB crystallin siRNA-transfected RPE cells treated with NaIO_3_. Furthermore, the PPAR_γ_ ligand A-PAF inhibited ROS production in αB crystallin knockdown RPE cells treated with NaIO_3_. This finding of ROS inhibition by PPAR_γ_ ligand in RPE cells is consistent with some recent reports in other cell types. In mesangial cells, PPAR_γ_ ligand rosiglitazone abolished ROS generation during exposure to high glucose, while inhibition of PPAR_γ_ by GW9662 caused ROS generation in normal glucose [41]. Further, αB crystallin was shown to effectively inhibit both ROS formation and apoptosis in cultured vascular endothelial cells [42]. The ROS-inhibitory function of PPAR_γ_ could arise from the antioxidative properties reported for PPAR_γ_. For example, it was shown recently that thiazolidinediones, synthetic ligands of PPAR_γ_, effectively protected pancreatic beta-cells from oxidative stress by an increase in the expression of the antioxidative enzyme catalase [43]. Similarly, antioxidative, neuroprotective function for PPAR_γ_ was reported in a model of Parkinson's disease [44]. It will be of interest to investigate whether the observed antiapoptotic function of PPAR_γ_ in RPE is linked to any changes in endogenous antioxidant enzymes. In this context, our laboratory has shown that overexpression of αB crystallin protects human RPE from oxidative and ER stress and upregulation of GSH and its biosynthetic enzymes are involved in this process [31], [32], [45].

The phosphoinositide 3-kinase (PI3K)-Akt pathway serves to coordinate the cellular response and ultimately determine cell fate [46]. Akt activation enhances RPE cell survival. It was reported that H_2_O_2_ induced PI3K and thereby activated Akt in human RPE cells [47]. AKT activation occurs through direct oxidation of phosphatase tensin homologue (PTEN) in acute oxidative stress [48]. We found in the present study that phosphorylated Akt and the signaling proteins downstream of AKT increased in RPE cells after treatment with NaIO_3_, Further, knockdown of αB crystallin by siRNA suppressed the activation of Akt. Together, these data suggest αB crystallin mediated protection of RPE cells from NaIO_3_ induced oxidative stress involves AKT. Working with HeLa cells, Pasupuleti et al. found evidence for activation of the PI3K/Akt cell survival pathway by alphaA crystallin by promoting phosphorylation of PDK1, AKT and PTEN [49]. It is of interest that Zhao et al reported that RPE dedifferentiation and hypertrophy in a model of oxidative phosphorylation (OXPHOS) deficiency or NaIO_3_ administration to B6 mice resulted in the stimulation of AKT/mammalian target of rapamycin (AKT/mTOR pathway [7]. Further, evidence for RPE oxidative damage and a rapid reduction of RPE65 and several other RPE-characteristic proteins was found [7]. This led the authors to suggest that mTOR pathway inhibition could be an effective therapeutic strategy for retinal degenerative diseases involving RPE stress [50].

In conclusion, our data show that αB crystallin plays a critical role in protection of NaIO_3_ induced oxidative and retinal degeneration in part through upregulation of AKT phosphorylation and PPAR_γ_ expression.

## Materials and Methods

### Ethics statement

This study conforms to applicable regulatory guidelines at the University of Southern California, principles of human subject protection in the Declaration of Helsinki and principles of animal research in the Association for Research in Vision and Ophthalmology and Statement for the Use of Animals in Ophthalmic and Vision Research. All procedures with mice were performed in compliance with the Keck School of Medicine Institutional Animal Care and Use Committee approved protocols and the ARVO Statement for the Use of Animals in Ophthalmic and Vision Research. The Institutional Review Board (IRB) approved our use of human RPE cells under protocol #HS-947005 (valid until April 4, 2014). Human fetal eyes (16–18 weeks of gestation) were obtained from advanced Bioscience Resources Inc. (ABR, Alameda, CA) and written informed consent was obtained from all donors. The University of Southern California Institutional Animal Care committee approved our animal studies under protocol #11710 (valid until October 18, 2014).

The 129S6/SvEvTac wild type mice were purchased from Taconic Farms (Germantown, NY), and the αB crystallin knockout mice in 129S6/SvEvTac background were obtained from the National Eye Institute [28], [29]. Mice aged between 6 and 8 weeks maintained on a standard laboratory chow in an air-conditioned room equipped with a 12-hour light/12-hour dark cycle were used in all studies.

### Experimental groups and NaIO_3_ treatment

The mice were divided into four groups of seven mice per group: control wild type (PBS-treated WT), NaIO_3_-treated wild type (NaIO_3_-treated WT), control (PBS) αB crystallin knockout mice, and NaIO_3_-treated αB crystallin knockout mice.

Experiments to determine the dose and time-dependent effect of NaIO_3_ were performed using doses of 20 mg/kg, 35 mg/kg, 50 mg/kg and 70 mg/kg body weight and duration of the study was one week to three weeks post NaIO_3_ administration. Briefly, varying doses of sodium iodate (NaIO_3_; Sigma, St. Louis, MO) diluted with Phosphate buffered saline (PBS) were injected through the tail vein to restrained mice. Animals injected with equivalent volumes of PBS served as controls. Electroretinography and fundus photograph (see below) were assessed 21 days post-injection. After the tests were performed, mice were euthanized with CO_2_ and their eyes processed for histology.

### Electroretinography (ERG)

Mice were dark-adapted overnight and anesthetized by intraperitoneal injection of ketamine (100 mg/kg body weight) and xylazine (10 mg/kg body weight). Pupils were dilated with topical administration of 2.5% phenylephrine containing 0.5% tropicamide, and the cornea was anesthetized with 0.5% proparacaine. Mesopic ERGs were measured using a non-attenuated light stimulus. To measure cone responses, a 6 lux white background light was delivered through the other arm of the coaxial cable to suppress rod responses, and a non-attenuated light stimulus was applied. *a*-Wave amplitude was measured from the baseline to the trough of the *a*-wave, while *b*-wave amplitude was measured from the trough of the *a*-wave to the peak of the *b*-wave [51].

### Fundus photography

Mice were anesthetized by administration of ketamine and xylazine as described above. Pupils were dilated and the cornea was anesthetized with 0.5% proparacaine. Images were captured using a 35 mm Kowa hand-held fundus camera (Genesis, Tokyo, Japan).

### Histopathologic analysis

Eyes were enucleated and the anterior segments were removed. The remaining posterior eye cups were snap-frozen in tissue freezing medium (Triangle Biomedical Sciences, Durham, NC), Optimal cutting temperature (OCT). Cryostat Sections (8 µm) were stained with hematoxylin and eosin (H&E), to assess the histopathologic changes.

Mouse retinal sections were scanned and retinal thickness was measured (Aperio ScanScope; Leica Biosystems) using Aperio software. Cell numbers in RPE layer, GCL layer, INL layer and ONL layer were determined by counting the nuclei in a 50 µm wide region of retinal section located at equal distance from the ora serrata and the optic disc. For each group, three eyes were dissected. For each, three different regions were counted by Image J 4.3.2 (NIH Image). Average cell numbers and standard deviation were calculated using Statlab (SPSS Inc, Chicago, Illinois, USA).

### Human and mouse RPE cell cultures

All procedures conformed to the Declaration of Helsinki for research involving human subjects and were performed with the approval of the institutional review board (IRB) of the University of Southern California. Human RPE cells were isolated from fetal human eyes of 16–18 wks gestation (Advanced Bioscience Resources, Inc., Alameda, CA) as previously described [52], [53]. Cells were cultured in DMEM (Fisher Scientific, Pittsburgh, PA) with 2 mM L-glutamine, 100 U/ml penicillin, 100 µg/ml streptomycin (Sigma, St. Louis, MO), and 10% heat-inactivated fetal bovine serum (FBS, Irvine Scientific, Santa Ana, CA). The preparations contained >95% RPE cells (cytokeratin-positive). Cells used were from passages 2 to 4. Primary mouse RPE cells were isolated as previously described [54]. Primary mouse RPE cells were isolated from 4 to 6 week old WT (129S6/SvEvTac) and αB crystallin knockout mice. RPE cells were cultured in DMEM containing 20% FBS and antibiotics until confluent and P3 cells were used for experiments.

### αB crystallin small interfering RNA (siRNA) transfection

Human RPE cells were switched to DMEM containing 0.5% FBS shortly before transfection. siRNA targeting αB crystallin was diluted in DMEM without serum. HiPerFect Transfection Reagent (Qiagen, Valencia, CA) was added to the diluted siRNA and mixed by vortexing. After incubation for 10 min at room temperature, the complexes were added dropwise to RPE cells. The final siRNA concentration was 5 nM. The cells were harvested or fixed for further assay 24 hours later. The sequence for siRNA targeting αB crystallin was: sense: r(CCA GGG AGU UCC ACA GGA A)dTdT; antisense:r(UUC CUG UGG AAC UCC CUG G) dTdT; nonsilencing control siRNA (scrambled siRNA): sense r(UUC UCC GAA CGU GUC ACG U) dTdT; antisense:r(ACG UGA CAC GUU CGG AGA A) dTdT. Forty-eight hours after transfection, *in vitro* effects of NaIO_3_ were studied either with a fixed final concentration 200 µg/ml added to the culture medium or at different doses as specified.

### Determination of ROS

To determine the compartmentalized generation of reactive oxygen species (ROS), mitochondria were labeled by a cell-permeable mitochondria-specific red fluorescent dye (MitoTracker, Molecular Probes); stained with carboxy-H2-DCFDA (Molecular Probes; 5 µM for 1 h at 37 °C), and rapidly evaluated by confocal microscopy (LSM510, Zeiss, Thornwood, NY, USA) as previously described [28], [31]. A yellow color is observed when ROS (green) are colocalized in the mitochondria (red). In some experiments, the effect of treatment with Azelaoyl PAF (A-PAF) (Sigma, St, MO, USA), a PPAR_γ_ ligand at a concentration of 20 µM and GW9662 (Cayman Chemicals, Ann Arbor, Mich, USA), a PPAR_γ_ antagonist, at a concentration of 10 µM was determined [55], [56].

### Determination of necrosis and apoptosis with NaIO_3_

Propidium Iodide (PI) stains DNA of necrotic cells [28], [31]. Human RPE cells on an eight-well Lab-TekTM chamber were treated with 10 mg/ml PI (Roche Applied Science) for 15 min at 25 °C in the dark. Cells were washed once with ice-cold PBS and observed under a laser scanning confocal microscope (LSM510, Zeiss, Thornwood, NY, USA).

Apoptosis (DNA fragmentation) was detected by the terminal deoxynucleotidyl transferase (TdT)-mediated dUTP-biotin nick end-labeling (TUNEL) method according to the manufacturer's protocol (In situ cell death detection kit-POD; Roche Applied Science). In short, after treatment with several doses of NaIO_3_ at room temperature, RPE cells on eight-well Lab-TekTM chambers were fixed in 1% paraformaldehyde solution and rinsed with PBS. Cells were then incubated with the TUNEL reaction mixture containing TdT and fluorescence UTP for 1 hour at 37°C in a humidified chamber. The nucleotides incorporated into DNA breaks were detected by applying anti-fluorescein peroxidase (POD) conjugate and peroxidase substrate.

### Immunocytochemistry of Cleaved Caspase-3

Human RPE cells on an eight-well Lab-TekTM chamber were fixed in 4% paraformaldehyde for 30 min, and then permeabilized using 0.2% Triton-X 100 at 37°C for 15 min. Blocking was achieved by addition of 1% goat serum for 20 min. The samples were incubated with primary anti-cleaved caspase-3 antibody (Cell Signaling; 1∶200) for 1 hr at room temperature. After washing with PBS, secondary biotinylated conjugated goat anti-rabbit antibody (1∶400; Vector, Burlingame, CA, USA) was applied to the slides for 30 min at room temperature. After washing with PBS, streptavidin peroxidase (Invitrogen, Camarillo, CA, USA) was applied to the slides for 30 min. 3-Amino-9-Ethylcarbazole (AEC) was added to the slide (AEC Substrate Kit, Invitrogen, Camarillo) which produced a red colored deposit. Sections were examined and photographed with microscope (Leica, Germany).

### Western blot analysis

Cells were lysed, supernatants were collected, and proteins were resolved on Tris-HCl 10% polyacrylamide gels (Ready Gel; Bio-Rad, Hercules, CA) at 120 V. The proteins were transferred to PVDF blotting membrane (Millipore, Bedford, MA). The membranes were probed with antibody for phospho-αB crystallin (Ser 59, Stressgen), pan-AKT (C67E4, Cell Signaling), phospho-AKT (Ser 473, Cell Signaling), phospho-PDK1 (Ser 241, Cell Signaling), phospho-c-Raf (Ser 259, Cell signaling), phospho-GSK-3β (Ser 9, Cell Signaling), PPAR_γ_ (Santa Cruz Biotechnology) all at 1∶1,000 dilution. Membranes were washed and incubated with a horseradish peroxidase (HRP)-conjugated secondary antibody (1∶3,000, Vector Laboratories, Burlingame, CA) for 30 min at room temperature. Images were developed by adding ECL chemiluminescence detection solution (Amersham Pharmacia Biotech, Cleveland, OH). Monoclonal anti mouse GAPDH was used as the loading control.

### Statistics

All experiments were performed at least three times. The data were analyzed using the Student's t-test (amplitudes of ERG; number of nuclei of outer nuclear layer, inner nuclear layer and ganglion layer histopathology; ROS; TUNEL; Cleaved caspase 3; and western blot) or Chi-square (fundus photography, RPE layer histopathology) and P<0.05 was considered as significant.

## Supporting Information

Figure S1 Fundus images showing time-dependent effect of a single dose of NaIO_3_ on wild type (WT) and αB crystallin -/- (αB-/-) mice. Representative images from a single mouse from WT and αB-/- groups are shown on the left accompanied by data for all experimental animals on the right. Fundus photography was taken one, two and three weeks after tail vein injection of PBS or 20 mg/kg NaIO_3_. PBS-treated WT and PBS-treated αB -/- did not exhibit any degenerative changes (data not shown). NaIO_3_- treated WT mice did not show retinal degeneration at any time point (A). However, NaIO_3_- treated αB-/- mice showed patchy retinal degeneration two and three weeks after NaIO_3_ injection (B). Arrow indicates the site of patchy retinal degeneration. αB-/- refers to αB crystallin knockout mice.
(TIF)

Figure S2 NaIO_3_-induced cell death in RPE layer of αB crystallin knockout mouse retina as determined by TUNEL staining. TUNEL staining was performed after WT and αB crystallin knockout mice were injected with 20 mg/kg NaIO_3_. No TUNEL+ cells were observed in the RPE layer of WT retina while TUNEL+ cells could be easily identified in the RPE layer of αB crystallin knockout retina (white arrow). Scale bar  = 50 µm.
(TIF)

## References

[1] Gehrs KM, Anderson DH, Johnson LV, Hageman GS (2006) Age-related macular degeneration-emerging pathogenic and therapeutic concepts. Ann Med. 38: 450–471. 17101537 10.1080/07853890600946724 PMC4853957

[2] Ambati J, Fowler B (2012) Mechanisms of Age-related macular degeneration. Neuron. 75: 26–39. 22794258 10.1016/j.neuron.2012.06.018 PMC3404137

[3] Bird AC (2010) Therapeutic targets in age-related macular degeneration. J Clin Invest. 120: 3033–3041. 20811159 10.1172/JCI42437 PMC2929720

[4] Klein ML, Ferris FL 3rd, Armstrong J, Hwang TS, Chew EY, et al (2008) Retinal precursors and the development of geographic atrophy in age-related macular degeneration. Ophthalmology. 115: 1026–1031. 17981333 10.1016/j.ophtha.2007.08.030

[5] Ramkumar HL, Zhang J, Chan CC (2010) Retinal ultrastructure of murine models of dry age-related macular degeneration. Prog Retin Eye Res 29: 169–90. 20206286 10.1016/j.preteyeres.2010.02.002 PMC2854213

[6] Enzmann V, Row BW, Yamauchi Y, Kheirandish L, Gozal D, et al (2006) Behavioral and anatomical abnormalities in a sodium iodate-induced model of retinal pigment epithelium degeneration,. Exp Eye Res 82: 441–448. 16171805 10.1016/j.exer.2005.08.002

[7] Zhao C, Yasumura D, Li X, Mathes M, Lloyd M, et al (2011) mTOR-mediated dedifferentiation of the retinal pigment epithelium initiates photoreceptor degeneration in mice. J Clin Invest 121: 369–383. 21135502 10.1172/JCI44303 PMC3007156

[8] Markovets AM, Saprunova VB, Zhdankina AA, Fursova AZh, Kolosova NG (2011) Alterations of retinal pigment epithelium cause AMD like retinopathy in senescence-accelerated OXYS rats. Aging (Albany, NY) 3: 44–54. 21191149 10.18632/aging.100243 PMC3047138

[9] Kolosova NG, Muraleva NA, Zhdankina AA, Stefanova NA, Fursova AZ, et al (2012) Prevention of age-related macular degeneration-like retinopathy by rapamycin in rats. Amer J Pathol 181: 472–477. 22683466 10.1016/j.ajpath.2012.04.018

[10] Malek G, Johnson LV, Mace ME, Saloupis P, Schmechel DE, et al (2005) Apolipoprotein E allele-dependent pathogenesis: a model for age related macular degeneration. Proc Natl Acad Sci USA 102: 11900–11905. 16079201 10.1073/pnas.0503015102 PMC1187976

[11] Ding JD, Johnson LV, Herrmann R, Farsiu S, Smith SG, et al (2011) Antiamyloid therapy protects against retinal pigmented epithelium damage and vision loss in a model of age-related macular degeneration. Proc Natl Acad Sci USA 108: E279–87. 21690377 10.1073/pnas.1100901108 PMC3136266

[12] Karan G, Lillo C, Yang Z, Cameron DJ, Locke KG, et al (2005) Lipofuscin accumulation, abnormal electrophysiology, and photoreceptor degeneration in mutant ELOVL4 transgenic mice: a model for macular degeneration. Proc Natl Acad Sci USA 102: 4164–4169. 15749821 10.1073/pnas.0407698102 PMC554798

[13] Zhu D, Wu J, Spee C, Ryan SJ, Hinton DR (2009) BMP4 mediates oxidative stress-induced cell senescence and is overexpressed in age-related macular degeneration. J Biol Chem 284: 9529–9539. 19158083 10.1074/jbc.M809393200 PMC2666605

[14] Zhu D, Deng X, Xu J, Hinton DR (2009) What determines the switch between atrophic and neovascular forms of age related macular degeneration? -the role of BMP4 induced senescence. Aging (Albany, NY). 12: 740–745. 10.18632/aging.100078 PMC2806048 20157553

[15] Longbottom R, Fruttiger M, Douglas RH, Martinez-Barbera JP, Greenwood J (2009) Genetic ablation of retinal pigment epithelial cells reveals the adaptive response of the epithelium and impact on photoreceptors,. Proc Natl Acad Sci USA 106: 18728–18733. 19850870 10.1073/pnas.0902593106 PMC2765920

[16] Kleinman ME, Kaneko H, Cho WG, Dridi S, Fowler BJ, et al (2012) Short-interfering RNAs induce retinal degeneration via TLR3 and IRF3. Mol Ther. 20: 101–108. 21988875 10.1038/mt.2011.212 PMC3255577

[17] Kaneko H, Dridi S, Tarallo V, Gelfand BD, Fowler BJ, et al (2011) DICER1 deficit induces AluRNA toxicity in age-related macular degeneration. Nature 471: 325–330. 21297615 10.1038/nature09830 PMC3077055

[18] Tarallo V, Hirano Y, Gelfand BD, Dridi S, Kerur N, et al (2012) DICER1 loss and Alu RNA induce age-related macular degeneration via the NLRP3 inflammasome and MyD88. Cell 149: 847–859. 22541070 10.1016/j.cell.2012.03.036 PMC3351582

[19] Franco LM, Zulliger R, Wolf-Schnurrbusch UE, Katagiri Y (2009) Decreased visual function after patchy loss of retinal pigment epithelium induced by low-dose sodium iodate,. Invest Ophthalmol Vis Sci. 50: 4004–4010. 19339739 10.1167/iovs.08-2898

[20] Ringvold A, Olsen EG, Flage T (1981) Transient breakdown of the retinal pigment epithelium diffusion barrier after sodium iodate: a fluorescein angiographic and morphological study in the rabbit,. Exp Eye Res 33: 361–369. 7297617 10.1016/s0014-4835(81)80088-7

[21] Kiuchi K, Yoshizawa K, Shikata N, Moriguchi K, Tsubura A (2002) Morphologic characteristics of retinal degeneration induced by sodium iodate in mice. Curr Eye Res 25: 373–379. 12789545 10.1076/ceyr.25.6.373.14227

[22] Redfern WS, Storey S, Tse K, Hussain Q, Maung KP, et al (2011) Evaluation of a convenient method of assessing rodent visual function in safety pharmacology studies: effects of sodium iodate on visual acuity and retinal morphology in albino and pigmented rats and mice. J Pharmacol Toxicol Methods 63: 102–114. 20619348 10.1016/j.vascn.2010.06.008

[23] Kannan R, Sreekumar PG, Hinton DR (2012) Novel roles for alpha crystallins in retinal function and disease. Prog Retin Eye Res 31: 576–604. 22721717 10.1016/j.preteyeres.2012.06.001 PMC3472046

[24] Nakata K, Crabb JW, Hollyfield JG (2005) Crystallin distribution in Bruch's membrane-choroid complex from AMD and age-matched donor eyes. Exp Eye Res 80: 821–826. 15939038 10.1016/j.exer.2004.12.011

[25] De S, Rabin DM, Salero E, Lederman PL, Temple S, et al (2007) Human retinal pigment epithelium cell changes and expression of alphaB-crystallin: a biomarker for retinal pigment epithelium cell change in age-related macular degeneration. Arch Ophthalmol. 125: 641–645. 17502503 10.1001/archopht.125.5.641

[26] Xi J, Farjo R, Yoshida S, Kern TS, Swaroop A, et al (2003) A comprehensive analysis of the expression of crystallins in mouse retina,. Mol Vis 9: 410–419. 12949468

[27] Fort PE, Lampi KJ (2011) New focus on alpha-crystallins in retinal neurodegenerative diseases. Exp Eye Res. 92: 98–103. 21115004 10.1016/j.exer.2010.11.008 PMC4454605

[28] Yaung J, Jin M, Barron E, Spee C, Wawrousek EF, et al (2007) alpha-Crystallin distribution in retinal pigment epithelium and effect of gene knockouts on sensitivity to oxidative stress. Mol Vis 13: 566–577. 17438522 PMC2652021

[29] Brady JP, Garland DL, Green DE, Tamm ER, Giblin FJ (2001) AlphaB-crystallin in lens development and muscle integrity: a gene knockout approach,. Invest Ophthalmol Vis Sci. 42: 2924–2934. 11687538

[30] Yaung J, Kannan R, Wawrousek EF, Spee C, Sreekumar PG, et al (2008) Exacerbation of retinal degeneration in the absence of alpha crystallins in an in vivo model of chemically induced hypoxia. Exp Eye Res. 86: 355–365. 18191123 10.1016/j.exer.2007.11.007 PMC2731668

[31] Dou G, Sreekumar PG, Spee C, He S, Ryan SJ (2012) Deficiency of alphaB crystallin augments ER Stress-induced apoptosis by enhancing mitochondrial dysfunction. Free Radic Bio Med. 53: 1111–1122. 22781655 10.1016/j.freeradbiomed.2012.06.042 PMC3454510

[32] Sreekumar PG, Spee C, Ryan SJ, Cole SPC, Kannan R, et al (2012) Mechanism of RPE cell death in alpha-crystallin deficient mice: A novel and critical role for MRP1-mediated GSH efflux. PLoS ONE. 7(3): e33420 10.137/journal.pone.0033420 22442691 PMC3307734

[33] Sreekumar PG, Kannan R, Kitamura M, Spee C, Barron E, et al (2010) AlphaB crystallin is apically secreted within exosomes by polarized human retinal pigment epithelium and provides neuroprotection to adjacent cells. PLoS ONE 5(10): e12578 doi:10.1371.journal.pone.0012578 20949024 10.1371/journal.pone.0012578 PMC2951891

[34] Ousman SS, Tomooka BH, van Noort JM, Wawrousek EF, O'Connor KC (2007) Protective and therapeutic role for alphaB-crystallin in autoimmune demyelination. Nature 448: 474–479. 17568699 10.1038/nature05935

[35] Brownell SE, Becker RA, Steinman L (2012) The protective and therapeutic function of small heat shock proteins in neurological diseases. Front Immunol. 3: 74. 22566955 10.3389/fimmu.2012.00074 PMC3342061

[36] Rothbard JB, Kumellas MP, Brownell S, Adams CM, Su L, et al (2012) Therapeutic effects of systemic administration of chaperone alphaB crystallin associated with binding proinflammatory plasma proteins. J Biol Chem 287: 9708–9721. 22308023 10.1074/jbc.M111.337691 PMC3322989

[37] Organisciak D, Darrow R, Gu X, Barsalou L, Crabb JW (2006) Genetic, age and light mediated effects on crystallin protein expression in the retina,. Photochem Photobiol 82: 1088–1096. 16602829 10.1562/2005-06-30-RA-599

[38] Sakaguchi H, Miyagi M, Darrow RM, Crabb JS, Hollyfield JG (2003) Intense light exposure changes the crystallin content in retina,. Exp Eye Res 76: 131–133. 12589783 10.1016/s0014-4835(02)00249-x

[39] Kase S, He S, Sonoda S, Kitamura M, Spee C, et al (2010) alphaB-crystallin regulation of angiogenesis by modulation of VEGF,. Blood. 115: 3398–3406. 20023214 10.1182/blood-2009-01-197095 PMC2858494

[40] Zhou P, Ye H-F, Jiang Y-X, Yang J, Zhu X-J, et al (2012) AlphaA crystallin may protect against geographic atrophy-meta analysis of cataract vs cataract surgery for geographic atrophy and experimental studies. PLoS ONE 7(8): e43173 10.1371/journal.pone.0043173 22916220 PMC3423426

[41] Whiteside C, Wang H, Xia L, Munk S, Goldberg HJ, et al (2009) Rosiglitazone prevents high glucose-induced vascular endothelial growth factor and collagen IV expression in cultured mesangial cells,. Exp Diabetes Res 2009: 910783. 19609456 10.1155/2009/910783 PMC2709725

[42] Liu B, Bhat M, Nagaraj RH (2011) AlphaB-crystallin inhibits glucose-induced apoptosis in vascular endothelial cells,. Biochem Biophys Res Commun 321: 254–258. 10.1016/j.bbrc.2004.06.151 15358243

[43] Chung SS, Kim M, Lee JS, Ahn BY, Jung HS (2011) Mechanism for antioxidative effects of thiazolidinediones in pancreatic beta-cells. Am J Physiol Endocrinol Metab 311: E912–11. 10.1152/ajpendo.00120.2011 21846907

[44] Martin HL, Mounsey RB, Mustafa S, Sathe K, Teismann P (2012) Pharmacological manipulation of peroxisome proliferator activator receptor gamma (PPAR gamma) reveals a role for anti-oxidant protection in a model of Parkinson's disease. Exp Neurol 235: 528–538. 22417924 10.1016/j.expneurol.2012.02.017 PMC3350857

[45] Sreekumar PG, Hinton DR, Kannan R (2012) Glutathione metabolism and its contribution to antiapoptotic properties of alpha-crystallins in the retina. In Studies on Retinal and Choroidal Disorders (Stratton RD, Hauswirth WW, Gardner TW, eds.Humana Press) Chapter 9, p. 181–202.

[46] Datta SR, Dudek H, Tao Y, Masters S, Fu H (1997) Akt phosphorylation of BAD couples survival signals to the cell-intrinsic death machinery. Cell 91: 231–241. 9346240 10.1016/s0092-8674(00)80405-5

[47] Yang P, Peairs JJ, Tano R, Jaffe GJ (2006) Oxidant-mediated Akt activation in human RPE cells,. Invest Ophthalmol Vis Sci 47: 4598–4606. 17003457 10.1167/iovs.06-0140

[48] Kim JW, Kang KH, Burrola P, Mak TW, Lemke G (2008) Retinal degeneration triggered by inactivation of PTEN in the retinal pigment epithelium. Genes Dev 22: 3147–3157. 18997061 10.1101/gad.1700108 PMC2593608

[49] Pasupuleti N, Matsuyama S, Voss O, Doseff AI, Song K, et al.. (2010) The anti-apoptotic function of human alphaA-crystallin is directly related to its chaperone activity. Cell Death and Disease. 1: , e31; doi: 10;1038/cddis.2010.3 10.1038/cddis.2010.3 PMC3032290 21364639

[50] Zhu C, Vollrath D (2011) mTOR pathway activation in age-related retinal disease. Aging 3: 346–347. 21483039 10.18632/aging.100303 PMC3117448

[51] Zhu X, Wu K, Rife L, Cawley NX, Brown B, et al (2005) Carboxypeptidase E is required for normal synaptic transmission from photoreceptors to the inner retina. J Neurochem. 95: 1351–1362. 16219026 10.1111/j.1471-4159.2005.03460.x

[52] Sonoda S, Spee C, Barron E, Ryan SJ, Kannan R, et al (2009) A protocol for the culture and differentiation of highly polarized human retinal pigment epithelial cells. Nat Protoc 4: 662–673. 19373231 10.1038/nprot.2009.33 PMC2688697

[53] Sonoda S, Sreekumar PG, Kase S, Spee C, Ryan SJ, et al (2010) Attainment of polarity promotes growth factor secretion by retinal pigment epithelial cells: relevance to age-related macular degeneration. Aging 2: 28–42. 10.18632/aging.100111 PMC2837203 20228934

[54] Kerur N, Hirano Y, Tarallo V, Fowler BJ, Bastos-Carvalho A, et al (2013) TLR-independent and P2X7-dependent signaling mediate Alu RNA-induced NLRP3 inflammasome activation in geographic atrophy. Invest Ophthalmol Vis Sci 54: 7395–7401. 24114535 10.1167/iovs.13-12500 PMC3825570

[55] Murata T, He S, Hangai M, Ishibashi T, Xi XP, et al (2000) Peroxisome proliferator-activated receptor-gamma ligands inhibit choroidal neovascularization,. Invest Ophthalmol Vis Sci 41: 2309–2317. 10892878

[56] Cheng HC, Ho TC, Chen SL, Lai HY, Hong KF, et al (2008) Troglitazone suppresses transforming growth factor beta-mediated fibrogenesis in retinal pigment epithelial cells. Mol Vis14: 95–104. PMC2254970 18253093

