# Supplementary figures and images for: Protection of Retina by αB Crystallin in Sodium Iodate Induced Retinal Degeneration
Source: PLoS One. 2014 May 29;9(5):e98275. doi: 10.1371/journal.pone.0098275 (PMC4038555; doi:10.1371/journal.pone.0098275)

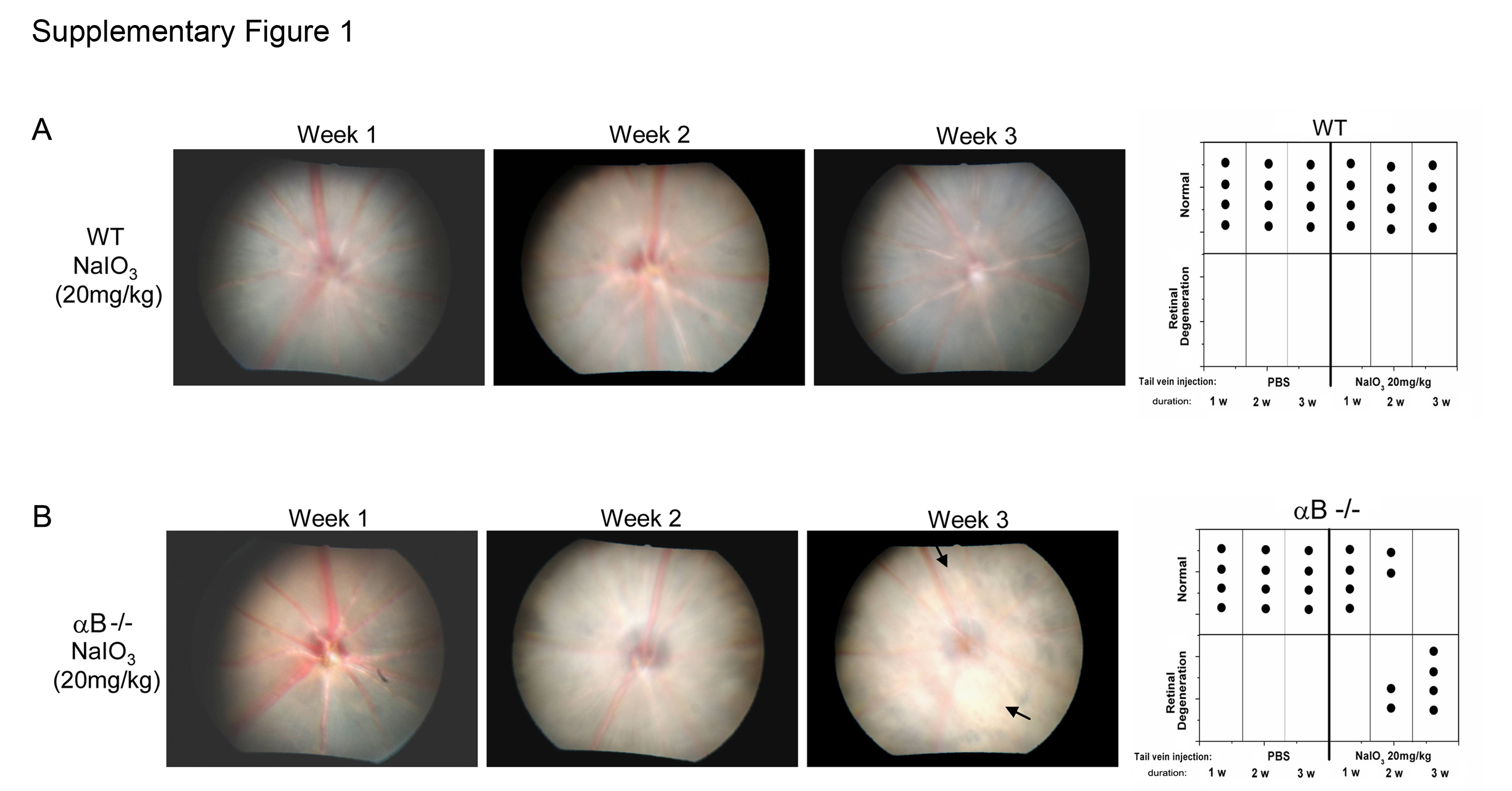

Supplement: Figure S1 — Fundus images showing time-dependent effect of a single dose of NaIO3 on wild type (WT) and αB crystallin -/- (αB-/-) mice. Representative images from a single mouse from WT and αB-/- groups are shown on the left accompanied by data for all experimental animals on the right. Fundus photography was taken one, two and three weeks after tail vein injection of PBS or 20 mg/kg NaIO3. PBS-treated WT and PBS-treated αB -/- did not exhibit any degenerative changes (data not shown). NaIO3- treated WT mice did not show retinal degeneration at any time point (A). However, NaIO3- treated αB-/- mice showed patchy retinal degeneration two and three weeks after NaIO3 injection (B). Arrow indicates the site of patchy retinal degeneration. αB-/- refers to αB crystallin knockout mice. (TIF) [file pone.0098275.s001.tif]

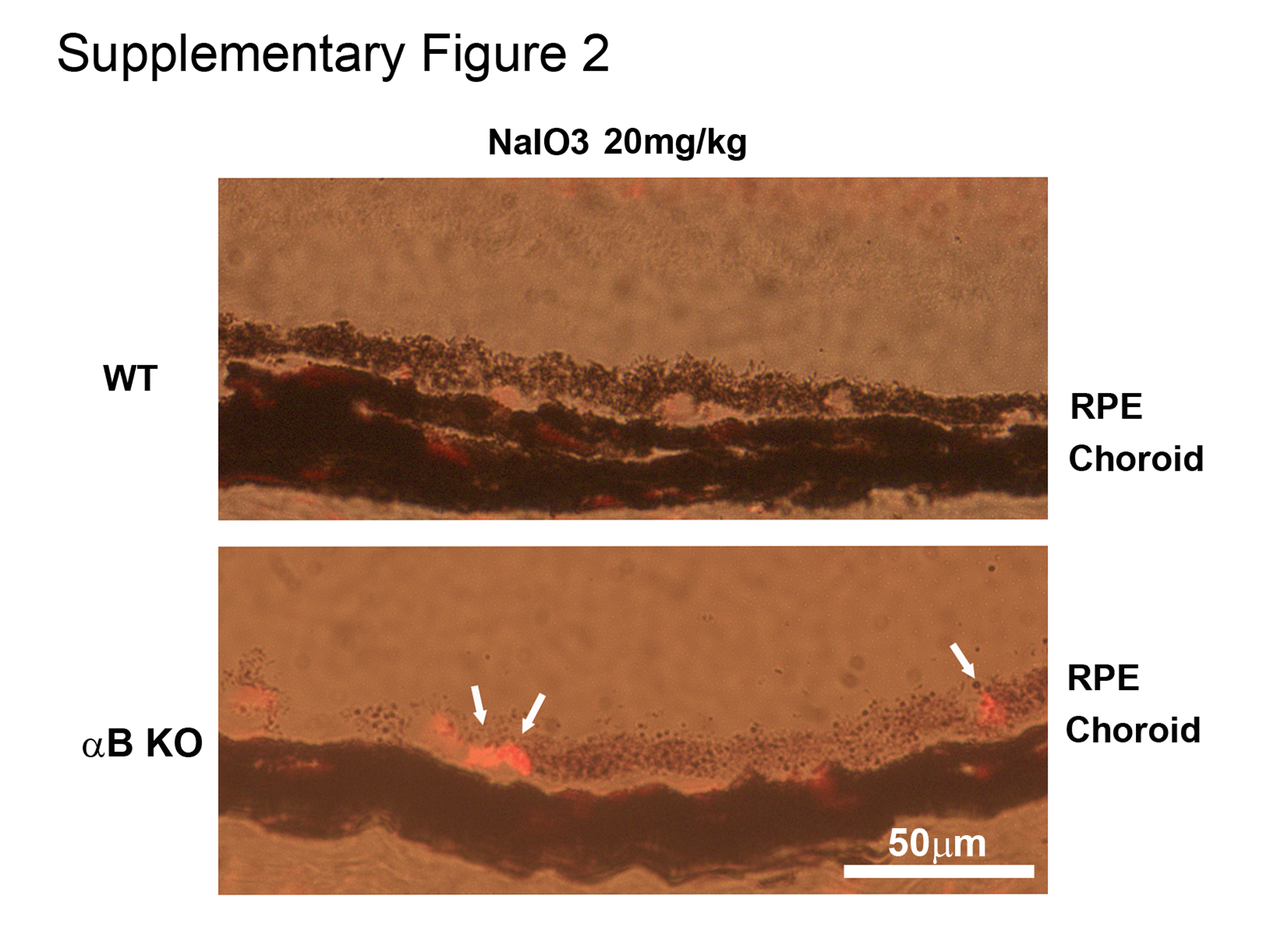

Supplement: Figure S2 — NaIO3-induced cell death in RPE layer of αB crystallin knockout mouse retina as determined by TUNEL staining. TUNEL staining was performed after WT and αB crystallin knockout mice were injected with 20 mg/kg NaIO3. No TUNEL+ cells were observed in the RPE layer of WT retina while TUNEL+ cells could be easily identified in the RPE layer of αB crystallin knockout retina (white arrow). Scale bar = 50 µm. (TIF) [file pone.0098275.s002.tif]
